# Vγ9Vδ2 T Cells as Strategic Weapons to Improve the Potency of Immune Checkpoint Blockade and Immune Interventions in Human Myeloma

**DOI:** 10.3389/fonc.2018.00508

**Published:** 2018-11-06

**Authors:** Barbara Castella, Assunta Melaccio, Myriam Foglietta, Chiara Riganti, Massimo Massaia

**Affiliations:** ^1^Laboratorio di Immunologia dei Tumori del Sangue, Centro Interdipartimentale di Ricerca in Biologia Molecolare, Università degli Studi di Torino, Turin, Italy; ^2^Dipartimento di Scienze Biomediche ed Oncologia Umana, Sezione di Medicina Interna ed Oncologia, Università degli studi di Bari “A. Moro”, Bari, Italy; ^3^SC Ematologia, AO S.Croce e Carle, Cuneo, Italy; ^4^Dipartimento di Oncologia, Università degli Studi di Torino, Turin, Italy

**Keywords:** Vγ9Vδ2 T cells, immune checkpoint blockade, immunotherapy, tumor vaccination, multiple myeloma

## Abstract

The advent of immune checkpoint (ICP) blockade has introduced an unprecedented paradigm shift in the treatment of cancer. Though very promising, there is still a substantial proportion of patients who do not respond or develop resistance to ICP blockade. *In vitro* and *in vivo* models are eagerly needed to identify mechanisms to maximize the immune potency of ICP blockade and overcome primary and acquired resistance to ICP blockade. Vγ9Vδ2 T cells isolated from the bone marrow (BM) from multiple myeloma (MM) are excellent tools to investigate the mechanisms of resistance to PD-1 blockade and to decipher the network of mutual interactions between PD-1 and the immune suppressive tumor microenvironment (TME). Vγ9Vδ2 T cells can easily be interrogated to dissect the progressive immune competence impairment generated in the TME by the long-lasting exposure to myeloma cellss. BM MM Vγ9Vδ2 T cells are PD-1^+^ and anergic to phosphoantigen (pAg) stimulation; notably, single agent PD-1 blockade is insufficient to fully recover their anti-tumor activity *in vitro* indicating that additional players are involved in the anergy of Vγ9Vδ2 T cells. In this mini-review we will discuss the value of Vγ9Vδ2 T cells as investigational tools to improve the potency of ICP blockade and immune interventions in MM.

## Introduction

Multiple myeloma (MM) is a disease characterized by the malignant growth of clonal plasma cells (hereafter referred to as myeloma cells) driven by intrinsic and extrinsic mechanisms. MM is uniformly preceded by a premalignant phase, termed monoclonal gammopathy of undetermined significance (MGUS). The risk of progression from MGUS to MM varies from 1 to 5% per year (1). Interestingly, myeloma cells isolated from the BM of MGUS already harbor many of the genetic and epigenetic abnormalities of myeloma cells isolated from patients with overt disease. Interestingly, long-term follow up has shown that almost 50% of high-risk MGUS never progresses to overt MM (2). These clinical data strongly support the concept that other factors, in addition to intrinsic myeloma cell features, are important to determine the fate and aggressiveness of myeloma cells.

The nature and relevance of the tumor microenvironment (TME) in MM have comprehensively been described elsewhere, including the role of immune cells (3, 4). We have anticipated these insights in the mid ‘80s, when we have shown a defective CD73 expression in CD8^+^ cells which was correlated with the proliferative activity of BM PC in both MGUS and MM (5). These initial findings have been corroborated by many other preclinical studies leading to the pioneeristic development of active specific immunotherapy approaches. The unique expression of idiotype (Id) by clonal B cells encouraged the generation of a variety of Id-specific vaccines (from protein- to DNA-based vaccines) which were able to induce long-lasting and tumor-specific immune responses (6).

Clinical results in allo-transplanted MM patients have strengthened the perception that the only chance to permanently eliminate residual myeloma cells [including those surviving high dose melphalan and autologous stem cell transplantation (ASCT)] is the recognition and elimination by allogeneic immune effector cells (7). The development of immunomodulatory imide drugs (IMiDs) and the clinical results obtained with lenalidomide (including maintenance treatment after ASCT) have brought further evidences that immune cells in the TME are key targets to interrupt the myeloma cell prosurvival network (8).

**Figure 1:**
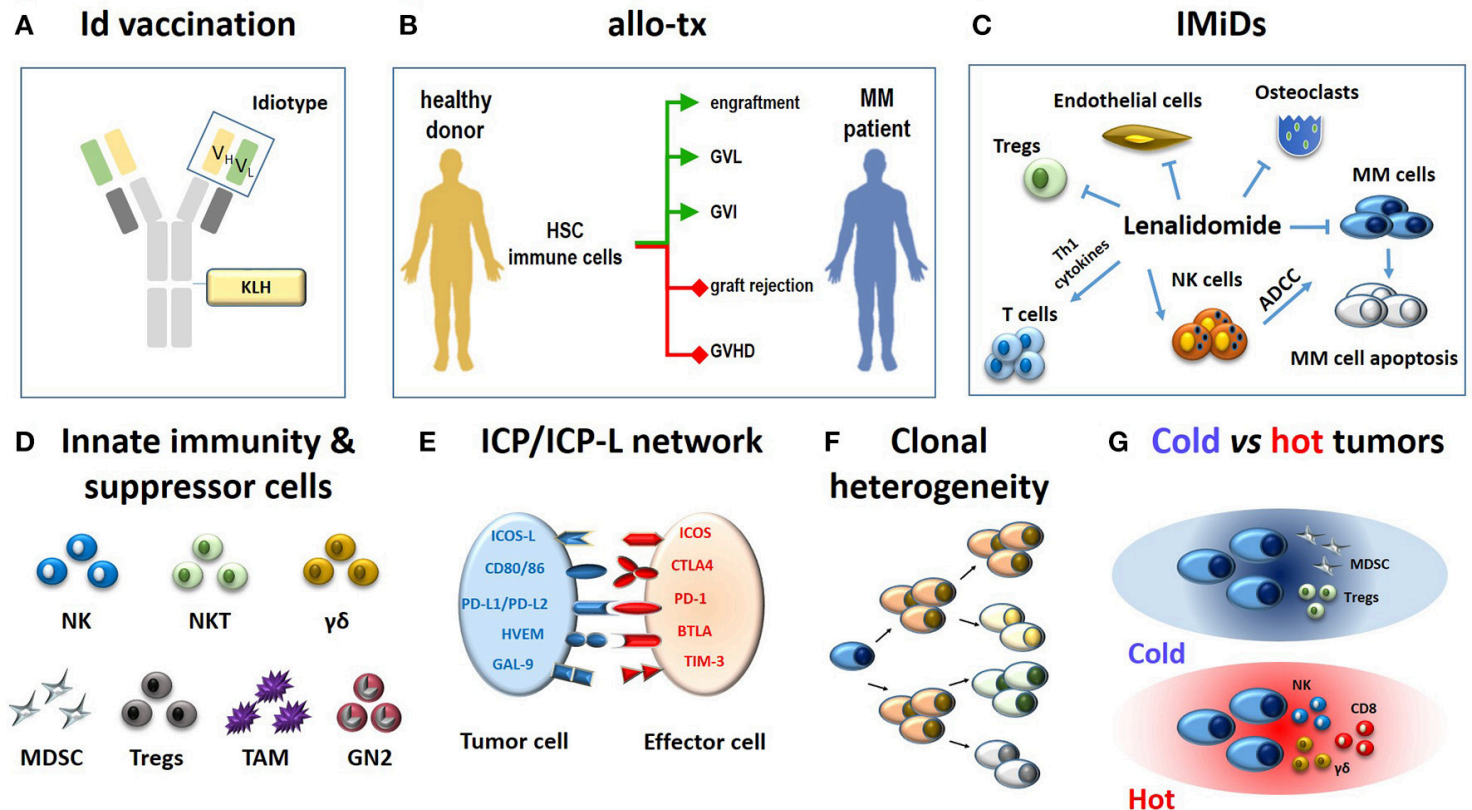
Immune-based approaches in MM patients **(A–C)** and major hurdles to their definitive clinical success **(D-G). (A)** The monoclonal immunoglobulin produced by myeloma cells is a very specific TAA. The antigenic determinants localized in the complementary determining regions of monoclonal heavy and light chains (yellow and green rectangles) are termed idiotypes (Id) and are tumor-specific. Id specifities have been used to address tumor-specific immune responses. A vaccine formulation consisting of Id-specific proteins conjugated with KLH as immunogenic carrier has been shown to generate very specific and long-lasting anti-myeloma immune responses (6). **(B)** The ultimate goals of allogeneic transplantation (allo-tx) are to ensure a rapid blood engraftment mediated by donor HSC and concurrently address donor immune effector cells to eliminate residual malignant cells (GVL) and to control post-transplant infections (GVI; green lines). Ideally, these goals are achieved in the absence of graft rejection and/or GVHD (red lines) (72). So far, it has not been possible to clearly separate GVL from GVHD in the clinical practice. **(C)** Immunomodulatory drugs like lenalidomide fine-tune multiple immune functions in MM patients: (i) they enhance and potentiate the cytototoxic and ADCC activity of T cells and NK cells, respectively; (ii) they inhibit myeloma cell growth and induce apoptosis; (iii) they inhibit osteoclasts, ECs, and Tregs suppressor functions. **(D)** The role of innate effector cells such as NK cells, NKT cells and γδ T cells has been neglected when initial immunotherapy approaches have been developed; the need to overcome or neutralize the suppressor role of MDSCs, Tregs, TAMs, and suppressive neutrophils type II (GN2) was unkown and not addressed. **(E)** Another major hurdle is represented by the ICP/ICP-L immune suppressive circuitry. The interactions between ICP expressed by effector cells (ICOS, CTLA-4, PD-, BTLA, TIM-3) and ICP-L expressed by myeloma cells and bystander cells in the TME (ICOS-L, CD80/CD86, PDL-1/PDL-2, HVEM, GAL-9) impair anti-myeloma immunity. **(F)** MM is characterized by clonal and subclonal diversity which is shaped over time by repeated treatments, responses, and relapses. This clonal heterogeneity facilitates the immune escape of myeloma cells. **(G)** The TME immune infiltration discriminates between cold and hot tumors. The former are characterized by the local recruitment and/or activation of immune suppressor cells like Tregs and MDSC; the latter are characterized by the presence of cytotoxic cells (NK, CD8, γδ T cells). Clonal diversity, mutational load, and treatments are key factors to drive the immune infiltration of cold vs. hot tumors. Hot tumors are more sensitive to immunotherapy than cold tumors. The MM TME is closer to cold than hot tumors. Id, idiotype; KLH, keyhole limpet hemocyanin; allo-tx, allogeneic transplantation; HSC, hematopoietic stem cells; MM, multiple myeloma; graft-vs.-leukemia, GVL; graft-vs.-infections, GVI; graft-vs. host desease (GVHD); IMIDs, immunomodulatory drugs; Tregs, regulatory T cells; MDSC, myeloid derived suppressor cell; tumor-associated macrophages (TAM); GN, granulocyte neutrofils; NK, natural killer; ADCC, antibody-dependent-cellular-cytotoxicity; NKT, natural killer T cells; ICP, immune checkpoint; ICP-L, immune checkpoint ligands; TAA: tumor associated antigen.

These approaches have significantly impacted on the clinical outcome, but none of them has generated such an impressive cure rate to definitely change the natural history of the disease (Figure 1).

## Reconsidering the immune competence of MGUS and MM patients

The unsatisfactory results of immune-based approaches in MM should not generate a pessimistic view. The reasons are rooted in the increased knowledge about the pathogenesis of the disease, the pathophysiology of immune responses, and the innovative technologies available to monitor the disease, assess clinical responses, and develop novel strategies of immune interventions. Additional progresses have been made by shedding some misconceptions like the wisdom that MGUS are immunologically blessed conditions in which myeloma cells are hold in check by very effective immune responses. This misconception was based on mouse models and preclinical results obtained in humans when much less was known about the mechanisms of immune surveillance and immune escape (9). Only recently, this misconception has been breached by us and others revealing that multiple immune dysfunctions are already present in MGUS (10–13).

Another misconception to be abandoned is that the remission state after ASCT represents a unique opportunity for immune interventions since it is possible to achieve a minimal residual disease (MRD) condition in this setting. We have shown more than 10 years ago that the T-cell receptor (TCR) repertoire is highly disrupted in patients in remission after ASCT (14). These results have been confirmed and consolidated (15) explaining why Id vaccination could not fulfil clinical expectations and why lenalidomide maintenance, even nowadays, significantly extends progression free survival (PFS), but does not definitely protect MM patients from late or very late relapse (8).

The time is ripe to apply more informative assays to investigate the immune competence of MGUS and MM. The aim of this minireview is to recapitulate how interrogating the immune competence of BM Vγ9Vδ2T cells has deepen our knowledge about the immune derangement occurring in MGUS and MM patients and how these informations can be applied to design more effective immune interventions in MM.

## Vγ9Vδ2 T cells as ultrasensitive tools to assess the immune suppressive TME commitment in MGUS and MM

Vγ9Vδ2 T-cells are non-conventional T cells half-way between adaptive and innate immunity with a natural inclination to react against malignant B cells, including malignant myeloma cells (16). These cells are able to sense supra-physiological concentrations of phosphorylated metabolites (pAgs) generated in the mevalonate (Mev) pathway of mammalian cells. Isopentenyl pyrophosphate (IPP) is the prototypic pAg recognized by Vγ9Vδ2 T cells. The pAgs-reacitivity of Vγ9Vδ2 T cells can be tested *in vivo* and *in vitro* by stimulating monocytes or dendritic cells (DC) with aminobisphosphonates like pamidronate or zoledronate (ZA). Both compounds inhibit farnesylpyrophosphate synthase in the Mev pathway (17, 18) and induce intracellular IPP accumulation and extracellular IPP release that are detected by Vγ9Vδ2 T cells. IPP recognition by Vγ9Vδ2 T cells is mediated by the γδ TCR in association with the isoform A1 of the butyrophilin-3 (BTN3A1) protein family (19, 20).

Vγ9Vδ2 T cells are endowed with peculiar functional properties which make them very good candidates for immunotherapy: they do not require MHC restriction and co-stimulation; they produce pro-inflammatory cytokines (IFN-γ and TNF-α); they recognize antigens shared by a variety of stressed and tumor cells; they behave as professional antigen-presenting cells (21); they can provide help to B cells to produce antibodies (22); and they can induce DC maturation boosting αβ T cell priming and MHC-restricted antigen-specific T-cell responses (23). We believe that this multifaceted array of immune functions gives a unique predisposition to Vγ9Vδ2 T cells to behave as very sentitive biosensors of the immune suppressive TME commitment occurring in the BM of MGUS and MM patients (24).

We have previously shown in a large series of patients (MGUS: *n* = 10; MM at diagnosis: *n* = 70; MM in remission: *n* = 52; MM in relapse: *n* = 24) that BM MM Vγ9Vδ2 T cells are unable to properly react to pAgs stimulation in terms of proliferation, CD107 expression and IFN-γ production. This is an early and long-lasting immune dysfunction, already detectable in MGUS individuals, largely anticipating that of CD8+ T cells and not disappearing even when most of tumor cells have been cleared by ASCT as in MM in remission. The investigation of pAgs reactivity of BM MM Vγ9Vδ2 T cells has been instrumental to show that the frequency of immune suppressor cells in the TME [bone marrow stromal cells (BMSC), regulatory T cells (Tregs) and myeloid-derived suppressor cells (MDSC)] are similar in the BM of MGUS, MM at diagnosis and MM in remission.

## Role of immune checkpoints (ICP) and ICP-ligands (ICP-L) in the immune suppressive TME commitment of MGUS and MM patients

Immune checkpoints (ICP) are key regulators of immune activation, immune homeostasis, and autoimmunity driven by interactions with the corresponding ligands (ICP-L) expressed by surrounding cells (25). In cancer, the ICP/ICP-L network is often hijacked by tumor cells to suppress anti-tumor immune responses. This has led to the development of anti-ICP/ICP-L monoclonal antibodies (mAbs) to treat a variety of cancers with heterogenous results.

Among the ICP/ICP-L pairs identified so far, the PD-1/PD-L1 axis plays a major role in the generation of the immune suppressive TME in MM. PD-L1 expression in myeloma cells is higher in MM and SMM than in MGUS and predicts an increased risk of disease progression (26, 27). Paiva et al. have shown a significant upregulation of PD-L1 expression in residual myeloma cells of MM patients who are in first complete remission (27). PD-L1 expression can protect residual myeloma cells from the immune modulation driven by lenalidomide and promote their immune escape and regrowth. Beside myeloma cells, MDSC, and BMSC also express high levels of PD-L1 cells in the BM microenvironment [24 and our unpublished data], underlining a redundancy of immune suppressor cells exploiting the ICP/ICP-L circuitry to hamper anti-myeloma immunity in the TME.

PD-L1 expression is paired by PD-1 overexpression in CD4^+^ and CD8^+^ T cells, and NK cells (28–30) isolated from PB and BM of MM patients creating a very effective network to protect myeloma cells from immune recognition and killing. Preliminary data from our laboratory indicate that multiple ICP can be expressed by effector cells, as already reported by Koyama's group in solid tumors (31).

These and other pre-clinical evidences (30, 32, 33) have been the groundwork to introduce anti-PD-1/PD-L1 treatment in MM patients, but clinical results have not met clinical expectations (34–36). These data have confirmed the complexity of the ICP/ICP-L and shown that single PD-1/PD-L1 blockade is insufficient to recover anti-tumor immune responses in MM patients. Investigating the defective pAg reactivity of BM MM Vγ9Vδ2 T cells represent a unique opportunity to identify potential partners and strategies to improve the efficacy of ICP/ICP-L blockade and immune interventions in MGUS and MM.

## Lessons from BM MM Vγ9Vδ2 T cells

The unsatisfactory results of anti-PD-1/PD-L1 monotherapy have stimulated the hunt for combinatorial treatment including lenalidomide (28, 37), elotuzumab (anti-SLAMF7) (38), histone deacetylase inhibitors, oncolytic reovirus (39), and radiation therapy (40). Lenalidomide and pomalidomide in combination with pembrolizumab (anti-PD-1) and dexamethasone have progressed up to phase III first-line trials, but unexpected toxicity in the pembrolizumab arm has led to the temporary discontinuation of these trials (https://www.onclive.com/web-exclusives/fda-discloses-data-on-halted-pembrolizumab-myeloma-trials). These hitches are paradigmatic examples how difficult is to carry on immunotherapy studies without a full knowledge about the TME landscape and the local conundrum of tumor-host interactions.

We have shown that a significant fraction of Vγ9Vδ2 T cells that are anergic to pAg stimulation in the TME of MGUS individuals and MM patients are PD-1+ (24). The attempts to fully recover anti-myeloma BM Vγ9Vδ2 T-cell activity *in vitro* by single PD-1 blockade has failed (24). Investigating the mechanisms of resistance to PD-1 blockade in PD-1^+^ BM MM Vγ9Vδ2 T cells can provide useful hints to improve the potency of ICP blockade in MM and other diseases.

**Figure 2:**
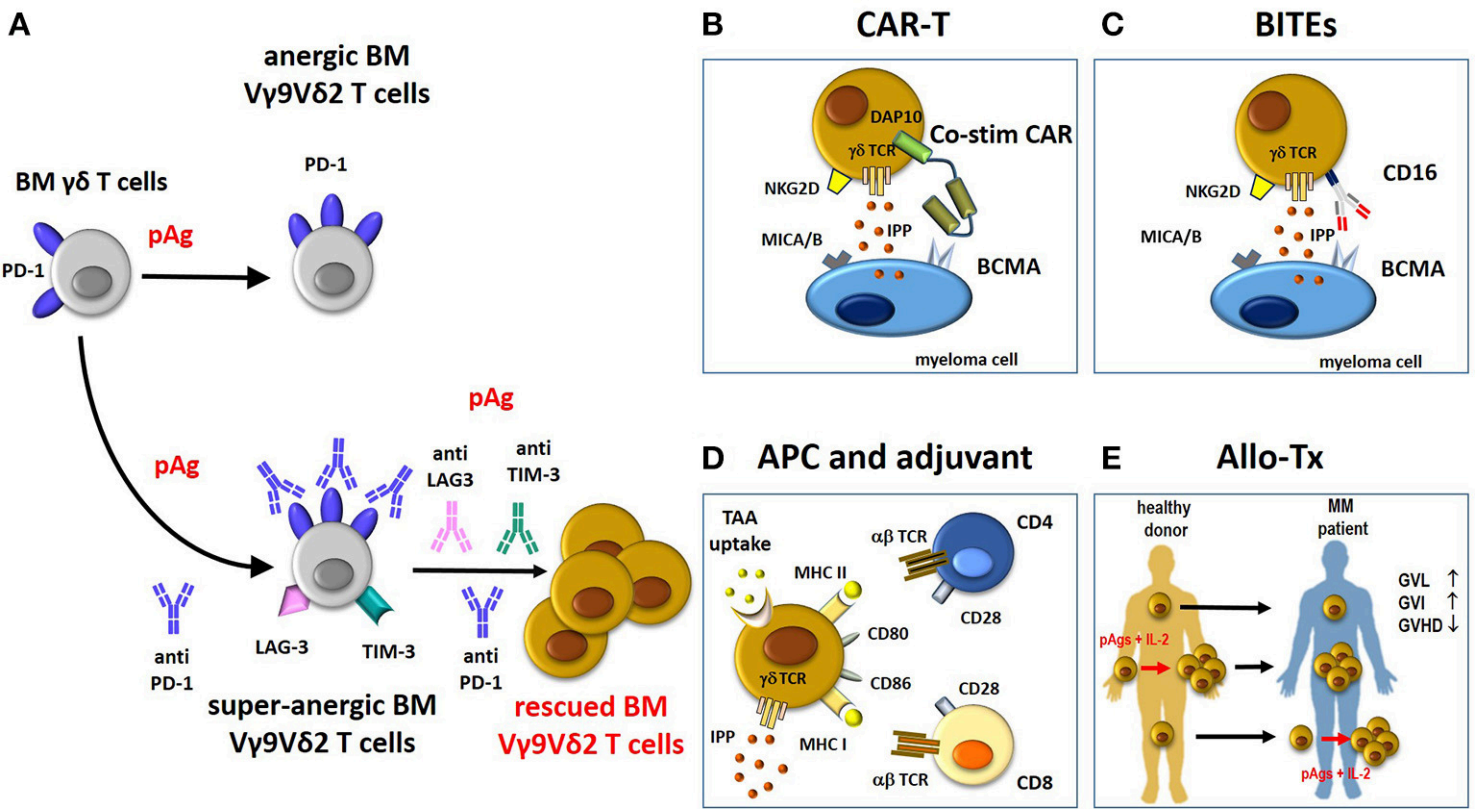
Potential contribution of rescued Vγ9Vδ2 T cells to immune treatments in MM. **(A)** BM Vγ9Vδ2 T cells in the TME are PD-1^+^ and anergic to pAg-stimulation. This anergy is not overcome by pAg stimulation in the presence of anti-PD-1. Paradoxically, the pAg + anti-PD-1 combination deepens the anergy of BM MM Vγ9Vδ2 T cells (super-anergic state). One possible hallmark of super-anergic immune effector cells is the expression of multiple ICP (i.e., LAG-3. TIM-3) on the same cells. Combinations of multiple anti-ICP antibodies (anti-PD-1/anti-LAG3/anti-TIM-3) is necessary to overcome the super-anergic state. If rescued from the anergic or super-anergic state, Vγ9Vδ2 T cells can become attractive candidates for immune interventions as proposed in panels B-E. Compared to conventional T cells, Vγ9Vδ2 T cells are not MHC-restricted and can be activated by pAgs (IPP), and stress-induced self ligands (i.e., MICA/B) other than CARs, tumor-specific transferred αβ *TCR* genes, and BITEs (see also text). **(B)** The “costimulatory only” CAR Vγ9Vδ2 T cells approach (58). In these cells, the cytotoxic capacity of CD3 (signal 1) is mediated through the native γδ-TCR recognition of IPP, whereas costimulation (signal 2) is provided by a CAR recognizing BCMA with an endodomain consisting of the innate NKG2D signaling molecule, DAP10. The cytotoxic ability of CAR Vγ9Vδ2 T cells is improved by the recognition of other molecules expressed by myeloma cells like MICA/B via NKG2D. **(C)** BITEs can be used to re-direct CD16-expressing Vγ9Vδ2 T cells against MM antigen (i.e., BCMA) and enhance their cytotoxic anti-tumor activity. **(D)** Vγ9Vδ2 T cells can act as APC to present TAA to MHC-restricted CD4+ and CD8^+^ T cells. APC-Vγ9Vδ2 T cells express many APC-related cell surface receptors like MHC-I and II, and co-stimulatory proteins (CD80, CD86). **(E)** The beneficial activity of Vγ9Vδ2 T cells in the allo-tx settings can be exploited as follows: i) direct infusion of unmanipulated grafts containing small amounts of donor circulating Vγ9Vδ2 T cells; these cells can increase in number after infusion depending on several factors like infections etc; (ii) donor Vγ9Vδ2 T cells from healthy donors are expanded *ex-vivo* before reinfusion in graft recipients using pAg like zoledronic acid and IL-2 (73); (iii) donor Vγ9Vδ2 T cells are expanded *in vivo* in the recipient after allo-tx with pAgs like zoledronic acid and IL-2. BM, bone marrow; pAg, phosphoantigen; CAR-T, chimeric antigen receptor-T; co-stim-CAR, “costimulatory only” CAR; BITEs, bispecifc T-cell engager; APC, antigen-presenting cell; TAA, tumor-associated-antigen; allo-Tx, allogenic-transplantation; GVHD, graft-vs.-host disease; GVL, graft-vs.-leukemia; GVI, graft-vs.-infections; IL-2, interleukin-2.

Multiple ICP expression by immune cells, paired by multiple ICP-L expression in tumor cells and surrounding cells in the TME is emerging as a general mechanism of cancer resistance to ICP blockade. Our preliminary results show that BM MM Vγ9Vδ2 T cells express multiple ICP engaged by the corresponding ICP-L expressed by myeloma cells and bystander cells. ICP-L overexpression in MDSC reinforces their intrinsic immune suppressive commitment, but ICP-L overexpression in endothelial cells and BMSC reflects a contranatural protumoral recruitment operated by myeloma cells in the TME. Our data showing that anergic PD-1^+^ Vγ9Vδ2 T cells up-regulate PD-1 and express alternative ICP (TIM3, LAG3; that we have defined super-anergic state), if stimulated with pAgs in the presence of single PD-1 blockade, indicates that the TME is reprogrammed to resist any mild and/or insufficient attempt to recover antitumor immune function (Figure 2). This is not very different from what we have learned from chemotherapy when polychemotherapy has replaced single-agent chemotherapy (i.e., ABVD for Hodgkin's disease, R-CHOP for diffuse large B-cell lymphoma, ICE for acute myeloid leukemia etc).

Currently, the most common strategies to overcome the onset of alternative ICP are combinations of multiple anti-ICP antibodies. This approach, supported by *in vitro* and *in vivo* data, is impeded by the prohibitive costs and increased side effects and toxicity in the clinical setting. The analysis of the molecular interactions between different ICP (PD-1, TIM-3, LAG-3) in anergic Vγ9Vδ2 T cells could help to identify mechanistic interventions to prevent alternative ICP uregulation and boost the immune potency of ICP inhibitors.

## Potential contribution of Vγ9Vδ2 T cells to novel immune treatments

The spectrum of immune interventions has significantly broadened in MM over the last few years thanks to novel findings and technical advances. Immune responses mediated by non-conventional T cells like Vγ9Vδ2 T cells, NKT cells, and CD1a-restricted T cells have gained significant consideration similar to MHC-restricted immune responses mediated by CD8^+^ cells. The characterization of suppressor cells like MDSC, Tregs, BMSC, and very importantly, the discovery of the ICP/ICP-L network have been other important steps to promote the renaissance of immunotherapy in MM. The identification of additional targets other than Id has led to an unprecedented surge of mAbs directed against myeloma cells (CD38, CD138, SLAMF7, CD138, BCMA), the TME (ICP/ICP-L), or both (CD38, SLAMF7, anti-PD-L1) (41, 42). Notably, CD38-targeted therapy with daratumumab has emerged as of the most effective passive immunotherapy ever developed in MM (43).

Current adoptive immunotherapy approaches under preclinical or clinical investigation include *ex-vivo* (CAR-T, TCR-engineered T cells) or *in vivo* redirected T cells [bispecifc T-cell engager (BiTEs)] (44, 45). Clinical trials testing BCMA-redirected CAR-T cells are producing impressive results in heavily pretreated relapsed and/or refractory MM patients (44–49).

TCR-engineered T cells are genetically modified in order to express αβ TCR with enhanced affinity for selected TAA. In contrast to CAR, αβ*TCR* gene transferred cells retain HLA restriction of Ag recognition and are sensitive to intracellular peptides (44, 45). Cancer testis antigens are under investigation as potential TAA in MM patients (50, 52).

Despite a growing enthusiasm, immunotherapy progresses are still facing many hurdles. The majority of MM treated with anti-CD38 mAbs (daratumumab) eventually progress and the mechanisms involved in resistance to daratumumab are largely unknown. CAR T cells also are not free from handicaps like reduced expression of BCMA on myeloma cells, short persistence or loss *in vivo* of functional CAR T cells (44–49). Bispecific CAR T cells targeting simultaneously two myeloma associated antigens may compensate the decreased BCMA expression, but it may also increase on-target off-tumor toxicity. MHC down-regulation on tumor cells may compromise the therapeutic efficacy of αβ*TCR* gene transferred T cells, whereas the eventual recognition of cross reactive epitopes from alternative target antigens may account for considerable on-target off-tumor toxicity. Autoimmune fatal complications have occurred with MAGE-A3 enhanced affinity αβ*TCR* gene transferred T cells (51). Another drawback of αβ*TCR* gene transfer to conventional CD3^+^ αβ T cells is the formation of mixed TCR dimers with unknown specificities due to pairing of endogenous and introduced α and β TCR chains (53).

BM Vγ9Vδ2 T cells can be very attractive candidates to deliver antitumor responses in MM, provided that they are rescued from the immune dysfunction they are afflicted. These cells recognize a broader range of targets (including metabolic targets like IPP and self-induced stress ligands) and possess a more favorable safety profile than conventional T cells (16). This unique feature has been exploited to reduce the potential “off target” toxicity of CAR Vγ9Vδ2 T cells (54–57). Fisher et at (58) have designed “costimulatory only” CAR Vγ9Vδ2 T cells in which activation signals 1 and 2 are provided by separate receptors. In these dual-receptor CAR Vγ9Vδ2 T cells, the cytotoxic capacity of CD3 (signal 1) is mediated via the native γδ-TCR recognizing IPP, whereas costimulation (signal 2) is provided by a CAR-mediated recognition of TAA mediated by DAP10, the endodomain consisting of the NKG2D receptor (Figure 2B). Normal healthy tissues which do not express IPP do not activate Vγ9Vδ2 TCR and are spared from Vγ9Vδ2 T cell cytotoxicity. Interestingly, these “costimulation only” CAR Vγ9Vδ2 T cells express lower levels of PD1 and TIM3 than traditional CAR Vγ9Vδ2 T cells after long term culture (58).

Vγ9Vδ2 T cells are excellent candidates for αβ*TCR* gene transfer without the risk of expression of undesired mixed TCR dimers (59). Another interesting approach is to engineer αβ T cells to express tumor-specific Vγ9Vδ2 TCRs (TEGs) to redirect αβ T cells against cancer cells (60). Vγ9Vδ2 TCR-redirected αβ T cells very efficiently kill cancer cell lines *in vitro* and primary acute myeloid leukemia blasts in a humanized mouse model. Very recently, TEGs have also been generated in MM patients and shown to be able to recognize and kill myeloma cells in a 3D model (61). Vγ9Vδ2 T cells can also be redirected against myeloma cells with BITEs (Figure 2C). The bispecific antibody [(HER2)2xCD16] has been used to re-direct CD16^+^ Vγ9Vδ2 T cells against Her2^+^ tumor cells that were killed with very high efficiency (62). HLA-independent recognition of TAA by tumor-redirected CAR Vγ9Vδ2 T cells or BITEs-activated Vγ9Vδ2 T cells may prelude to the development of allogeneic “off the shelf” CAR products.

Another unique feature of Vγ9Vδ2 T cells is their capacity to act as antigen-presenting cells (APC) to boost antigen-specific immune responses mediated by CD8^+^ cells (21, 63) (Figure 2D). Combination therapy of Vγ9Vδ2 T-APC-based vaccines with ICP blockade may have synergistic activity leading to enhanced anti-tumor immune responses and long-lived immuno-surveillance (64, 65). These adjuvant properties are not lost even after chimerization of Vγ9Vδ2 T cells as demonstrated by Capsomidis A. (57)

Lastly, the multifunctional properties of Vγ9Vδ2 T cells may also be beneficial in the allo-tx setting (allo-tx) (Figure 2E) (66). Vγ9Vδ2 T cells have been reported to cause less graft-vs.-host disease (GVHD) than αβ T cells while retaining graft-vs.-leukemia activity (GVL) (67, 68). A protective effect of Vγ9Vδ2 T cells against both leukemia cell regrowth and infections has been reported in haploidentical HSCT depleted of TCR-αβ/CD19 lymphocytes (69). Lastly, recent studies suggest an overall favorable effect of high Vγ9Vδ2 T cells immune reconstitution after HSCT; patients with elevated numbers of Vγ9Vδ2 T cells had a significantly higher overall survival rate and a decreased rate of acute GVHD compared to patients with low Vγ9Vδ2 T cell counts (70).

## Conclusions

Investigation of BM MM Vγ9Vδ2 T cells has been useful to gather a faithfully picture of the immune suppressive TME in MGUS and MM. Understanding the mechanisms that are responsible for BM Vγ9Vδ2 T-cell dysfunction, with special regard to resistance to PD-1 blockade, can help to overcome ICP resistance and safely integrate ICP/ICP-L blockade in the immune treatments of MGUS and MM patients. The use of nanotechnologies may improve delivery of antagonistic antibodies to block ICP inhibitory receptors compared to free antibodies and improve T cell activation (71).

Finally, the functional rescue of BM Vγ9Vδ2 T cells is an attractive opportunity to exploit their multifaceted immune functions to carry on *ex-vivo* and *in vivo* adoptive immunotherapy interventions.

## Author contributions

All authors have made a substantial contributions to text and figures and have approved the manuscript for submission

### Conflict of interest statement

The authors declare that the research was conducted in the absence of any commercial or financial relationships that could be construed as a potential conflict of interest.
